# A standardized protocol for the detection of arboviruses in different *Aedes* mosquito species in North Borneo Sabah, Malaysia

**DOI:** 10.1016/j.mex.2025.103549

**Published:** 2025-08-06

**Authors:** Adlar Ryan Ngiam, Saiful Anuar Ju Ahmad, Mohd Farid Alias, Mohd Arshil Moideen, Norsyahida Sulaiman, Song-Quan Ong, Eric Chong Tzyy Jiann, Ping-Chin Lee, Sylvia Daim, Jodi M. Fiorenzano, Noel Cote, Andrew G. Letizia

**Affiliations:** a Malaysian Armed Forces (MAF), Malaysia; b Institute for Tropical Biology and Conservation, Universiti Malaysia Sabah, Jalan UMS, 88400 Kota Kinabalu, Sabah Malaysia; c Faculty of Science and Natural Resources, Universiti Malaysia Sabah, Jalan UMS, 88400 Kota Kinabalu, Sabah, Malaysia; d Faculty of Medicine & Defence Health, National Defence University Malaysia, Malaysia; e Biotechnology Research Institute, Universiti Malaysia Sabah, Jalan UMS, 88400 Kota Kinabalu, Sabah, Malaysia; f Faculty of Medicine and Health Sciences, Universiti Malaysia Sabah, Kota Kinabalu, Sabah, Malaysia; g US. Naval Medical Research Unit INDO PACIFIC, Singapore; h Vysnova Partners, LLC, Alexandria, VA 22314, USA

**Keywords:** RNA extraction, *Aedes* mosquitoes, Multiplex real-time PCR, Disease surveillance, Dengue, Zika, Chikungunya, Vector control

## Abstract

Arboviruses, including dengue, Zika and chikungunya viruses, are mainly transmitted by *Aedes* mosquitoes and pose a threat to public health. The viruses are transmitted by the primary vector, *Aedes aegypti*, which is more commonly found in urban environments. However, with increasing urbanization, the overlap of rural and forested areas where different *Aedes* species are found could also contribute to transmission. Nevertheless, most extraction methods focus on human blood samples or on *Ae. aegypti*, which limits standardization of virus detection in a variety of less common *Aedes* populations, especially sylvatic species.

In this study, we demonstrated a standardized protocol for extracting sufficient amounts of RNA for detection from a single mosquito sample. We validated the protocol by extracting arboviruses from six different *Aedes* species collected in the field in Sabah, Malaysia: *Ae. aegypti, Ae. albopictus, Ae. poecilus, Ae. butleri, Ae. niveus*, and *Ae. vexans*. Multiplex real-time PCR detection yielded consistent cycle threshold (Ct) values across species (range: 18.9–40.3), with a positivity cut-off of Ct < 41. Our results show that this protocol improves current practice by extending the target sample to different *Aedes* mosquito species, ultimately contributing to more efficient virus detection and supporting more comprehensive surveillance, even in ecologically diverse environments.

## Graphical abstract

(include separately in a different file – TIFF format)

**Figure:**
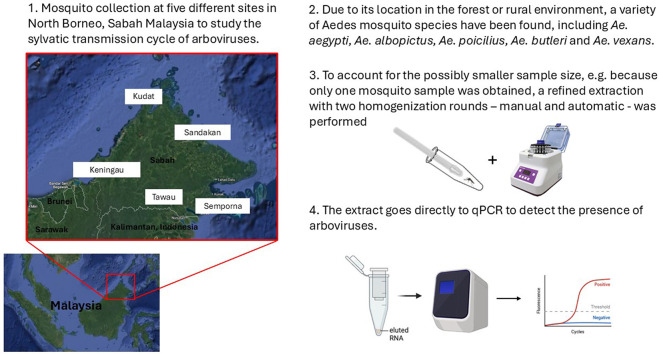

## Specifications table

**Table:** 

**Subject area**	Medicine and Dentistry
**More specific subject area**	Mosquito-borne diseases, pathogen surveillance, PCR
**Name of your protocol**	An optimized protocol for the identification of arboviruses in different *Aedes* mosquitoes
**Reagents/tools**	1. Qiagen QIAamp DSP Virus Spin Kit 2. Smith Scientific Tissue Grinding Pestle 3. 70 % ethanol 4. Vortex mixer 5. Legend Micro 21 Centrifuge 6. BioRad CFX-96 Real-Time PCR
**Experimental design**	The experimental design investigated the potential vectors of arboviruses in forest areas, particularly for sylvatic transmission of dengue virus. *Aedes* mosquitoes were collected from rural areas and forests in North Borneo, Sabah, Malaysia. After collection, mosquitoes were identified and sorted into the species including *Ae. aegypti, Ae. albopitcus, Ae. poecilus, Ae. butleri, Ae. niveus and Ae. vexans*. The standard methods for the detection of arboviruses from mosquitoes usually involve the extraction of RNA from a pool of mosquitoes to obtain a sufficient RNA yield. This can be somewhat challenging if the samples do not reach the desired number, e.g. for relatively sparsely collected species such as *Ae niveus*. In addition, standard methods usually focus on the extraction of human blood or the primary dengue vector usually *Ae. aegypti* or *albopictus*, which may be different from extracting RNA from other *Aedes* mosquitoes. In addition, commercially available kits such as the QIAamp DSP Virus Spin Kit are primarily intended for use with liquid samples (e.g. serum and plasma) and do not include digestion steps required for solid tissues such as mosquito samples. To address these limitations, we have adapted and optimized the protocol to include the critical homogenization steps required for effective lysis of mosquito tissue. While these changes differ from the kit’s original protocol, they are fundamental to successful RNA extraction from insect vectors. These modifications help ensure the applicability of the protocol to different *Aedes* species. We implemented a single-tube workflow that simplified sample handling and reduced processing time and risk of contamination. The mosquito samples were processed individually or in fours, depending on the specific trap used to collect them with the corresponding GPS information recorded. We validated the extraction protocol using multiplex qPCR, which detects Dengue, Zika and Chikungunya viruses and compared the results to standard extraction methods. This detection method is highly sensitive and allows for the confirmation of viral RNA even in small or degraded samples. The combination of RNA extraction and multiplex qPCR enables faster and more accurate detection compared to conventional methods, further highlighting the utility of our protocol for arbovirus monitoring.
**Trial registration**	Not applicable
**Ethics**	All authors confirm that we have complied with all relevant ethical regulations. This project was approved by the Malaysian Ministry of Health (NMRR ID –23–00,934- TOM), the Ethics Committee of Universiti Malaysia Sabah [JKEtika 3/23(13)] and the Animal Ethics Committee of UMS (AEC 007/2023). We obtained a permit from the Sabah Biodiversity Centre (SaBC) to collect mosquitoes in the forest area of Sabah [JKM/MBS.1000–2/2 JLD.16 (139)]
**Value of the Protocol**	1. RNA extraction was optimized for different species of Aedes mosquitoes, namely *Ae. aegypti, Ae. albopictus, Ae. poecilus, Ae. butleri, Ae. niveus and Ae. vexans*. 2. Validation of an RNA extraction method that can obtain a positive result from a very small number of mosquito samples, e.g. from only one mosquito sample. 3. Provides detailed methods for the detection of arboviruses that can be adapted for other studies related to zoonotic/sylvatic transmission by native *Aedes* mosquitoes in forest areas. 4. Provides an effective tool to assist in evaluating mosquito-borne diseases especially among less common Aedes mosquito species.

## Background

Mosquito-borne diseases are a significant public health challenge [1]. Arboviruses, such as dengue, Zika and chikungunya virus, are particularly concerning but remain neglected diseases [2,3]. These viruses are mainly transmitted by *Aedes aegypti* and *Aedes albopictus* in urban areas and have increased due to many factors, including urbanization and expansion of agricultural activities [4]. For example, a total of 123,133 dengue cases were reported in Malaysia in 2023, an increase of 86.3 % compared to the 66,102 cases in 2022 [5]. Additionally, Zika and chikungunya viruses have been reported in many countries in Southeast Asia [6]. Likely the increasing prevalence is related to the rapid urbanization process in the region, mainly driven by the increase in breeding sites of *Ae. aegypti* and the change in landscape from rural and forested to urban areas, which increases the exposure of native mosquito species to the human host [7]. For example, North Borneo, Sabah, Malaysia, which was mainly covered by forest, was rapidly urbanized or converted to agricultural land [8]. The relocation of the Indonesian capital from Jakarta to the west of BorneoIsland, Nusantara [9], and the hydropower megaproject in East and Central Borneo, Sarawak, Malaysia [10], were all associated with massive landscape changes and increased the exposure of native mosquito species harboring arboviruses for zoonotic and sylvatic transmission.

Research on mosquito-borne diseases in these areas is challenging because not only are the sampling areas usually difficult to reach [11], but most extraction and pathogen detection protocols focus on isolating and detecting the viruses from human blood [12,13] or from the primary vector - *Aedes aegypti*. Although standard techniques are available for *Ae aegypti* and *Ae albopictus*, there are still no standardized extraction and PCR detection methods for other *Aedes* species, especially for less common species found in North Borneo Sabah, Malaysia. In addition, previous protocols for the detection of arboviruses from mosquitoes usually require the extraction of RNA from a pool of mosquitoes to obtain a sufficient RNA yield. This can be somewhat challenging when samples do not reach the desired number, e.g. for relatively sparsely collected species such as *Ae niveus*.

Therefore, we aimed to optimize and standardize an extraction protocol capable of detecting arboviruses from different species of *Aedes* mosquitoes found in Sabah, Malaysia, minimizing the RNA requirement and enabling detection from a small number of samples, e.g. from only one mosquito sample. To obtain the RNA in a small number of samples, we implemented a rigorous cell lysis method that involves two stages of homogenization by humans and machines. This protocol was adapted to the Qiagen QIAamp DSP Virus Spin Kit (Qiagen, Germany), which we used to further improve RNA recovery to ensure high sensitivity for virus detection even with a relatively small number of samples. Our use of multiplex qPCR also enables the simultaneous detection of multiple viruses - including dengue, Zika and chikungunya viruses— - in a single run, further increasing efficiency and reducing costs. Overall, these optimizations contribute to a more efficient and robust method for monitoring arboviruses in diverse mosquito populations, providing valuable insights into disease ecology and transmission dynamics.

## Description of protocol

### Mosquito sampling

**Fig. 1:**
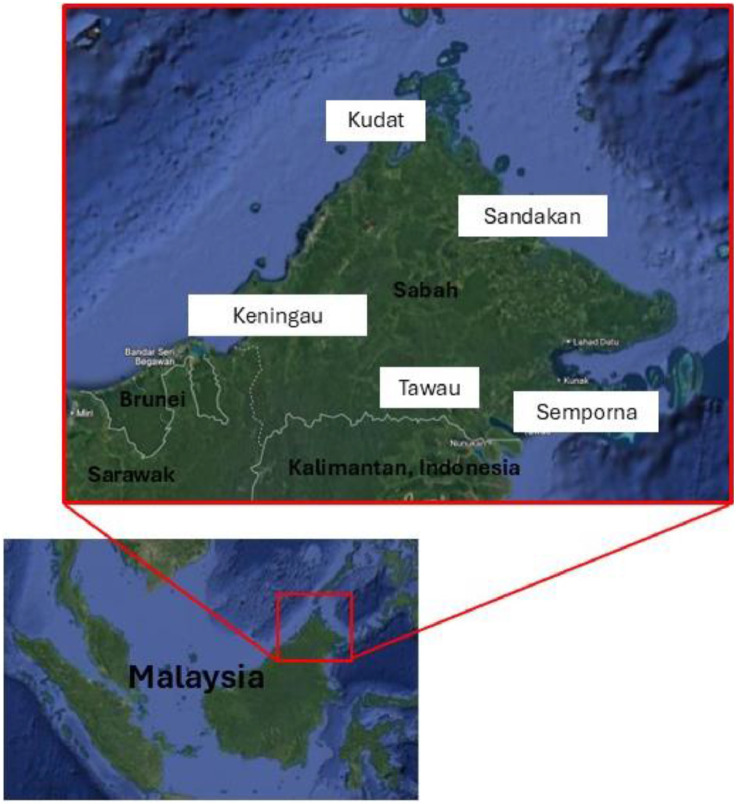
Mosquitoes sampling from five remote locations in North Borneo, Sabah Malasyia.

The sampling protocol was based on Ong et al. [11], using Centers for Disease Control and Prevention (CDC) light traps, Biogents-Sentinel (BGS), and human-baited double net (HDN) to collect mosquitoes from five remote locations in North Borneo, Sabah Malasyia, namely Semporna, Tawau, Sandakan, Kudat, and Keningau (Fig. 1). In brief, the mosquito samples from the CDC and BGS collection bags were transferred to a plastic container (9oz, 5 cm height), while for HDN, an aspirator was used to collect the mosquitoes that were between the inner and outer nets and put them into a plastic container. The plastic containers were labeled with the date, location, type of sampling and trap ID. The samples were stored at −20 °C to prepare them for pathogen screening.

### Extraction to multiplex real time PCR protocol

In the extraction protocol, the first round of extraction of a maximum of 25 samples took approximately 40 min (based on the TGrinder H24 tissue grinder used in this study). Briefly describe the process with the estimated time required. Mixing the mosquito samples with the buffer AL and the carrier RNA takes about 2 min. For cell lysis and RNA precipitation, the mixture was homogenized manually and automatically (2 to 4 min in total) and incubated at 56 °C for 15 min. In between, centrifugation and vortexing as well as further incubation (or precipitation) at room temperature took another 6 min. For RNA purification and elution, the process of adding lysate and buffer with further centrifugation takes about 10 more minutes, and the sample is ready for PCR testing after centrifugation.

### Sample preparation

The mosquito samples obtained from sampling were first sorted based on morphology according to taxonomic keys defined by Rattanarithikul (1982) and Jeffrey et al. (2023) to exclude non-*Aedes* mosquitoes. The samples were pooled according to the coordinates at which they were captured, in a labelled 1.5 mL microcentrifuge tube. The pooled samples ranged from 1 to 5 mosquitoes.

### Cell lysis and precipitation

Cell lysis was performed by adding 25 µL of Qiagen Protease (QP) into a Lysis Tube (LT) containing a mosquito sample. Subsequently, 200 µL of Buffer AL-carrier RNA (Table 1) was added, followed by glass beads, and the mixture was homogenized using a pestle. For manual homogenization, the samples were ground clockwise for 1 min and processed with the TGrinder H24 tissue grinder (Tiangen, China) for further homogenization and incubation. After incubating at 56 °C for 15 min in a heating block and periodic vortexing at 400 rpm, the LT tube was briefly centrifuged to remove drops. Then, 250 µL of ethanol was added, thoroughly mixed by vortexing, and the lysate was incubated at room temperature for 5 min. Following another brief centrifugation step, the tube was ready for RNA purification.

**Table 1:** Volume of Buffer AL-carrier RNA mix needed per batch of samples, samples (Qiagen, Germany).

No. of Samples	Vol. of Buffer AL (µL)	Vol. of Carrier RNA AVE (µL)
1	220	6.2
2	440	12.3
3	660	18.5
4	880	24.6
5	1100	30.8
6	1320	37.0
12	2640	73.9
18	3960	110.9
24	5280	147.8

### RNA purification

The entire lysate was carefully applied onto the MinElute column, avoiding wetting the rim, and centrifuged at 8000 rpm for 2 min. The MinElute column was then placed in a clean 2 mL Wash Tube (WT) and the WT containing the filtrate was discarded. This step was followed by the addition of 500 µL of Buffer AW1, centrifugation at 11,000 rpm for 1 min, replacement of the 2 mL WT, and then the WT containing the filtrate was discarded. The same process was repeated with 500 µL of Buffer AW2 and ethanol. Subsequently, the WT containing the filtrate was discarded, and the 2 mL WT was replaced before centrifuging at 14,000 rpm for 3 min to dry the membrane completely. To ensure complete drying, the assembly was incubated at 56 °C for 3 min.

### RNA elution

The MinElute column was placed in an Elution Tube, and the WT with filtrate was discarded. Buffer AVE was preheated at 60 °C for 1 min and applied to the center of the membrane. After incubating at room temperature for 5 min, centrifugation at 14,000 rpm for 2 min was performed, and the spin column was removed. The eluted RNA was then stored frozen until further use.

### Protocol validation and demonstration

To validate the protocol, we demonstrated the outcome of using the protocol by using two regions of Sabah (Fig. 1), using a total of 352 female of *Ae. aegypti* (20), *Ae. albopictus* (273) and *Ae. vexans* (25), *Ae. nivens* (10), *Ae. butleri* (24) from Tawau; a total of 127 of mixture of *Ae. aegypti* (45), *Ae. albopictus* (56), *Ae. vexans* (15), *Ae. butleri* (11). The ZIKV/DENV/CHIKV Real-Time PCR Kit (BioPerfectus, China) was used. A master mix was prepared for each batch of samples, with 20 µL per reaction, followed by the addition of 5 µL of RNA sample. Samples were kept on ice and protected from light. Subsequently, 20 µL of master mix was placed into each reaction tube or plate, and 5 µL of isolated RNA or controls (positive or blank) was added. Each run included at least one positive control and one blank (negative) control. The reaction tubes or plate were capped or sealed, then centrifuged to ensure all liquid settled at the bottom. Finally, the real-time PCR instrument was set up with the appropriate conditions (Table 2). Fluorescent signals were collected during this step through the FAM, VIC and ROX channels corresponding to dengue virus, Zika virus and chikungunya virus, respectively.

**Table 2:** PCR conditions of RNA amplification.

Step		Temperature	Time	Cycle
**1**	**Reverse transcription**	50 °C	10 min	1 cycle
**2**	**Initial denaturation**	95 °C	5 min	1 cycle
**3**	**Denaturation**	95 °C	10 s	45 cycles
	**Annealing, extension and fluorescent signal collection**	60 °C	30 s	

We assessed if the protocol could identify arboviruses from a variety of *Aedes* mosquitos using a total of 352 female of *Ae. aegypti* (20), *Ae. albopictus* (273), *Ae. vexans* (25), *Ae. nivens* (10), and *Ae. butleri* (24) from Tawau (Fig. 1) and a total of 127 of *Ae. aegypti* (45), *Ae. albopictus* (56), *Ae. vexans* (15), *Ae. butleri* (11) from Kudat. Table 3 shows the positive arboviral results from the salivary cells of six different *Aedes* species and the number of mosquitos used in each sample pool for extraction and PCR testing. According to the supplier's instructions, at least four mosquitoes should be used in a pool. However, due to the small number of mosquito species collected, e.g. *Ae. niveus* and *Ae. poecilus*, some of the pool samples contained less including some with only a single mosquito. To preserve the fidelity of the protocol, mosquitoes of the same species but collected at different times or locations could not be pooled due to the experimental design which aimed to understand virus infection rates at a specific times and locations. 41 pools of 352 female *Aedes* mosquitoes tested positive for arboviruses, including 32 positive dengue viruses, 11 chikungunya viruses, four co-infections of chikungunya and dengue viruses, and one co-infection of dengue and Zika virus. The results from Kudat showed that of the 127 *Aedes* mosquitoes, a total of 17 pools were positive for dengue virus, with one sample simultaneously infected with dengue and chikungunya viruses.

**Table 3:** The positive result of RNA extraction from the salivary cells of six different *Aedes* species and the maximum number of mosquitoes used for one sample pool for extraction and PCR testing.

Species	Maximum number of mosquito in one pool of sample	Number of pools run for PCR testing	No. of mosquitoes shows positive result		
			Dengue	Chikungunya	Zika
*Ae aegypti*	4	51	3	1	
*Ae albopictus*	4	111	32	10	1
*Ae* vexans	2	45	1		
*Ae nivens*	1	4	4		
*Ae butleri*	2	23	8	1	
*Ae. Poecilus*	1	10	1		

We compared our protocol with the modified Rapid Analyte Measurement Platform (RAMP®) (Response Biomedical Corporation, Burnaby, BC, Canada) [14] which uses fluorescently stained particles coated with anti-dengue virus antibodies that bind to dengue virus. Additionally, we compared several important aspects to the procedures published by Tang et al. [15], who performed RT-PCR in large *Ae. albopictus* mosquito pools, in terms of sample size, contamination risk, and estimated time for the process. Table 4 summarizes the comparison based on the sample size, handling steps, and estimated processing time.

**Table 4:** Comparison between the other extraction protocol and the protocol presented in this study.

Aspects	Extraction protocol		
	Rapid Analyte Measurement Platform (RAMP®) [14]^a^	Tang *et al*., 2020 [15]	Protocol of this study
**Target cell**	Human blood cells or *Aedes* aegypti	*Aedes albopictus*	Salivary cell of six different species of *Aedes* mosquito
**Sample Size**	A maximum pool size of 25 mosquitoes was used as recommended by the manufacturer.	Due to the aim of the study (detection of RNA from a large sample of mosquitoes) therefore at least 165 pooled samples were used	Works with individual mosquitoes
**Contamination Risk**	Higher due to multiple transfers	Higher due to multiple transfers	Lower with fewer handling steps
**Positive Detection Rate ( %)**^b^	80 %	–	100 %
**Estimated extraction time required for a sample**	More than two hours	Not mentioned, but based on the study, infected mosquitoes must be placed in a large pool of mosquito samples and begin homogenization with liquid nitrogen, from a simple simulation, the time required from sample preparation to elution could take more than an hour.	∼ 40 min

a RAMP, which we used in this study, is an antigen-based immunoassay that detects the NS1 antigen.

b The positive detection rate ( %) is based on the positive result of the 20 mosquito extracts from the RT-PCR of this protocol.

**Fig. 2:**
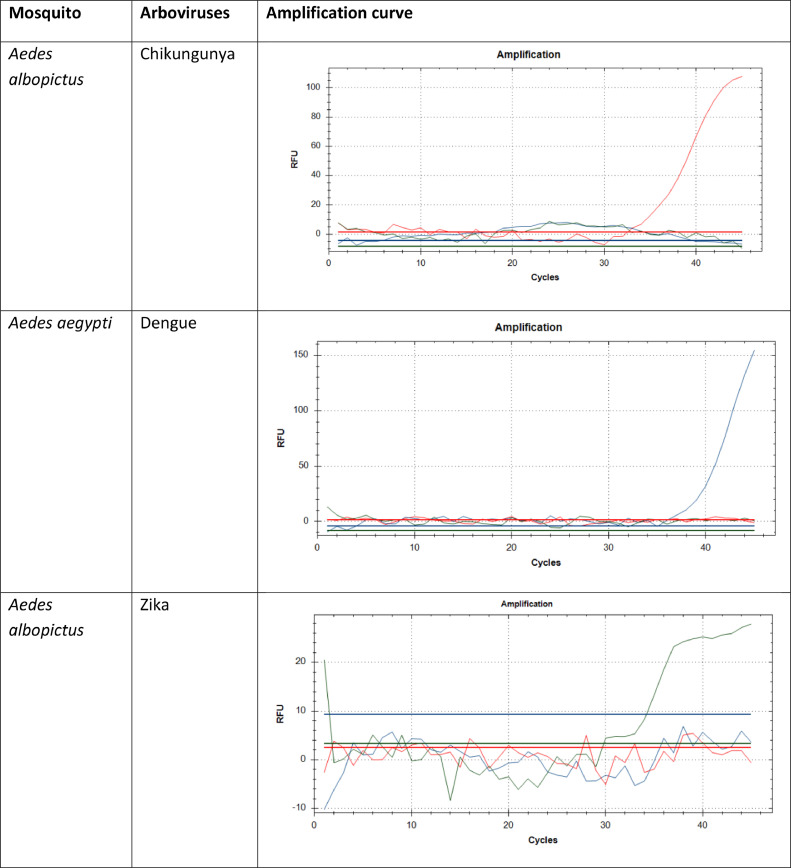

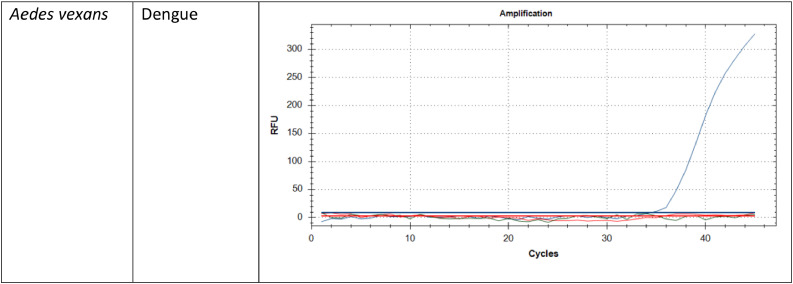
The positive results of some *Aedes* mosquito species with the amplification curve for the detection of arboviruses. The blue curve shows dengue virus, the green curve Zika virus and the red curve Chikungunya virus.

Fig. 2 shows some of the positive results of the amplification curve for the detection of arboviruses. In this study, qPCR was employed primarily as a screening tool to confirm the effectiveness of the extraction protocol. The Ct values observed across positive samples were consistent, indicating reliable extraction and reproducibility of results (Appendix). The results support the conclusion that the optimized extraction protocol effectively yields nucleic acids of sufficient quality for detection by qPCR.

**Appendix 1:** Result of the PCR for the samples collected in the inner forest of Tawau, depending on the trap and the date of capture.

Date	Trap*	Mosquito species	Dengue_virus	Chikungunya_virus	Zika_virus	Ct value
13/9/2023	CDC	*Ae albopictus*	.	Positive	.	31.29
23/9/2023	CDC	*Ae albopictus*	Positive	.	.	27.03
14/9/2023	CDC	*Ae albopictus*	.	Positive	.	32.58
29/9/2023	CDC	*Ae albopictus*	.	Positive	.	34.80
30/9/2023	CDC	*Ae albopictus*	Positive	Positive	.	20.10
14/9/2023	HDN	*Ae albopictus*	.	Positive	.	31.26
14/9/2023	CDC	*Ae albopictus*	.	Positive	.	30.56
21/9/2023	CDC	*Ae* vexans	.	Positive	.	38.89
12/9/2023	BGS	*Ae aegypti*	.	Positive	.	30.22
30/8/2023	HDN	*Ae* vexans	.	Positive	.	36.93
19/9/2023	HDN	*Ae albopictus*	Positive	.	.	18.97
19/9/2023	HDN	*Ae albopictus*	Positive	.	.	36.26
19/9/2023	HDN	*Ae albopictus*	Positive	.	.	35.83
19/9/2023	HDN	*Ae albopictus*	Positive	.	.	36.14
19/9/2023	HDN	*Ae albopictus*	Positive	.	.	36.36
19/9/2023	HDN	*Ae albopictus*	Positive	.	.	37.08
19/9/2023	HDN	*Ae albopictus*	Positive	.	.	35.62
19/9/2023	HDN	*Ae albopictus*	Positive	.	.	37.13
19/9/2023	HDN	*Ae albopictus*	Positive	.	.	34.95
23/9/2023	HDN	*Ae albopictus*	Positive	.	.	40.31
11/9/2023	HDN	*Ae albopictus*	Positive	.	.	36.51
30/9/2023	HDN	*Ae albopictus*	Positive	.	.	37.42
27/9/2023	HDN	*Ae albopictus*	Positive	Positive	.	36.99
25/9/2023	HDN	*Ae albopictus*	Positive	.	.	30.23
21/9/2023	HDN	*Ae albopictus*	.	Positive	.	22.90
17/9/2023	HDN	*Ae albopictus*	Positive	Positive	.	32.57
13/9/2023	HDN	*Ae albopictus*	Positive	.	.	30.72
12/9/2023	HDN	*Ae albopictus*	Positive	.	.	30.28
29/9/2023	HDN	*Ae albopictus*	Positive	Positive	.	36.60
26/9/2023	HDN	*Ae albopictus*	Positive	.	.	34.94
27/9/2023	HDN	*Ae albopictus*	Positive	.	.	36.11
13/9/2023	HDN	*Ae albopictus*	Positive	.	.	36.31
22/9/2023	HDN	*Ae albopictus*	Positive	.	.	36.01
28/9/2023	HDN	*Ae albopictus*	Positive	.	.	35.96
20/9/2023	CDC	*Ae albopictus*	Positive	.	Positive	39.32
24/9/2023	HDN	*Ae albopictus*	Positive	.	.	36.91

⁎ HDN – Human-baited Double Net; BGS - BG-Sentinel; CDC - Centers for Disease Control.

The protocol has some limitations. First, the extraction protocol utilizes a multiplex PCR that focuses on only three arboviruses: dengue, Zika and chikungunya. Other arboviruses could also be extracted, e.g. Japanese encephalitis virus (JEV), but were not investigated in this study. Multiplex PCR also suggests that the use of individual channels/probes may lead to different results, as each virus must be detected individually (which is a challenge with the single-tube strategy). Secondly, the multiplex kits did not cover the serotype, e.g. DENV 1 to 4 or genotype, which limits additional information.

## Author disclaimer

The views expressed in this article reflect the results of research conducted by the author and do not necessarily reflect the official policy or position of the Department of the Navy, Department of Defense, nor the United States Government.

## CRediT authorship contribution statement

**Adlar Ryan Ngiam:** Methodology, Software, Formal analysis, Investigation, Data curation, Writing – original draft, Writing – review & editing, Visualization. **Saiful Anuar Ju Ahmad:** Methodology, Investigation, Supervision, Project administration. **Mohd Farid Alias:** Conceptualization, Methodology, Supervision, Project administration. **Mohd Arshil Moideen:** Conceptualization, Methodology, Supervision, Project administration. **Norsyahida Sulaiman:** Methodology, Software, Validation, Formal analysis, Investigation, Data curation, Writing – review & editing. **Song-Quan Ong:** Conceptualization, Methodology, Formal analysis, Writing – original draft, Writing – review & editing, Visualization, Supervision, Project administration, Funding acquisition. **Eric Chong Tzyy Jiann:** Methodology, Software, Validation, Formal analysis, Investigation. **Ping-Chin Lee:** Methodology, Formal analysis, Writing – review & editing, Supervision, Project administration, Funding acquisition. **Sylvia Daim:** Conceptualization, Methodology, Supervision, Project administration. **Jodi M. Fiorenzano:** Methodology, Supervision, Project administration. **Noel Cote:** Methodology, Supervision, Project administration. **Andrew G. Letizia:** Supervision, Project administration, Funding acquisition.

## Declaration of interests

The authors declare that they have no known competing financial interests or personal relationships that could have appeared to influence the work reported in this paper.

All authors declare no competing or conflicts of interests. The views expressed in this article reflect the results of research conducted by the author and do not necessarily reflect the official policy or position of the Department of the Navy, Department of Defense, nor the United States Government. Jodi M. Fiorenzano (LCDR, MSC, USN), Noel Cote (LCDR, MSC, USN), Andrew G. Letizia (CAPT, MC, USN) are military service members. This work was prepared as part of my official duties. Title 17 U.S.C. 105 provides that `copyright protection under this title is not available for any work of the United States Government.' Title 17 U.S.C. 101 defines a U.S. Government work as work prepared by a military service member or employee of the U.S. Government as part of that person's official duties.

## Data Availability

Data will be made available on request.
